# *Lactobacillus plantarum* NDC 75017 alleviates the learning and memory ability in aging rats by reducing mitochondrial dysfunction

**DOI:** 10.3892/etm.2014.2000

**Published:** 2014-10-01

**Authors:** XINYAN PENG, JIONG MENG, TAO CHI, PENG LIU, CHAOXIN MAN, SHAOMIN LIU, YING GUO, YUJUN JIANG

**Affiliations:** 1 National Research Centre of Dairy Engineering and Technology, Northeast Agricultural University, Harbin, Heilongjiang 150028, P.R. China; 2 Key Laboratory of Dairy Science, Ministry of Education, Department of Food Science, Northeast Agricultural University, Harbin, Heilongjiang 150030, P.R. China; 3 Synergetic Innovation Center of Food Safety and Nutrition, Harbin, Heilongjiang 150030, P.R. China; 4 College of Food Engineering, Ludong University, Yantai, Shandong 264025, P.R. China

**Keywords:** *Lactobacillus plantarum* NDC 75017, cerebral cortex, mitochondrial dysfunction, aging, rat

## Abstract

The aim of the present study was to investigate the protective effect of *Lactobacillus plantarum* NDC 75017 on D-galactose (D-gal)-induced mitochondrial dysfunction in the rat cerebral cortex. Fifty rats were randomly divided into five groups (n=10 in each group). The rats in the aging model group were subcutaneously injected with 100 mg/kg D-gal and those in the protective groups were additionally orally administered *L. plantarum* NDC 75017 at doses of 1×10^8^, 1×10^9^ or 1×10^10^ CFU/100 mg body weight/day, respectively. The control rats were administrated an equal volume of the vehicle. Following continuous treatment for seven weeks, the learning and memory abilities and mitochondrial ultrastructure, function and adenosine triphosphate (ATP) levels were examined. The results showed that the learning and memory abilities and mitochondrial levels of ATP were significantly decreased in the D-gal-induced aging model group compared with those in the control group (P<0.01). In addition, marked changes in the mitochondrial functions and ultrastructure were observed between the groups. Seven weeks of *L. plantarum* NDC 75017 and D-gal coadministration significantly improved the learning and memory abilities of the rats compared with the D-gal-induced aging model group. Furthermore, the combination regime significantly improved the mitochondrial ultrastructure and functions, including the mitochondrial respiratory chain, mitochondrial membrane potential and mitochondrial permeability transition. The results revealed that the *L. plantarum* NDC 75017 was able to alleviate learning and memory injuries in aging rats by reducing the mitochondrial dysfunction induced by D-gal.

## Introduction

Mitochondrial dysfunction plays a major role in the process of aging and in aging-related neurodegenerative disorders. This is due to the crucial role of mitochondria in producing adenosine triphosphate (ATP), the main source of cellular energy (1–4). Furthermore, mitochondria are the target organelles for reactive oxygen species (ROS), which are a major source of physiologically produced oxidative stress during aging (3). A number of studies have demonstrated that mitochondrial dysfunction is closely associated with several aging-related diseases, including Alzheimer’s disease, Huntington’s disease (HD) and Parkinson’s disease (PD) (5–7). It has been confirmed that mitochondrial protection and the consequent reduction of oxidative stress are important targets for the prevention and treatment of the early stages of these aging-related diseases (8,9). D-galactose (D-gal) is a natural reducing sugar in the body that is normally metabolized by D-galactokinase and galactose-1-phosphate uridyltransferase in animals. An excess of D-gal results in abnormal metabolism (10). The progressive deterioration in learning and memory skills, as well as the production of ROS in the brain tissue of rodents, has been previously reported in the literature (11). It has been shown that the administration of D-gal for 6–10 weeks induces mimetic aging changes in the brain tissue of rats. This has been utilized to establish animal models in studies investigating potential therapies and prevention strategies for certain age-associated diseases (12–15).

In recent years, the most frequently used antioxidant food supplements have included certain lactic acid bacteria (LAB) and medicinal plants. It has been revealed that several dietary supplements, including spinach and citrus fruits extracts, may be beneficial in protecting against age-related neurological disorders (16,17). A number of LAB strains have the ability to scavenge free radicals, improve the activity of antioxidant enzymes and inhibit lipid oxidation. Hathout *et al* (18) demonstrated that treatments with *Lactobacillus casei* or *Lactobacillus reuteri* protected rats fed an aflatoxin-contaminated diet from oxidative stress. Bay *et al* (19) reported that a skimmed-milk culture of LAB reduced lipid peroxidation in rat livers and brains. The abnormal expression of γ-aminobutyric acid (GABA), an important neurotransmitter in the brain, is implicated in the pathogenesis of anxiety and depression (20). A previous study showed that chronic administration of D-gal markedly decreased the number of GABA-immunoreactive neurons in the cortical layers of rats with D-gal-induced aging, which further contributed to their behavioral deficits (21). This is one of the suggested mechanisms by which LAB regulates brain function (22). In a previous study, the LAB strain *Lactobacillus plantarum* NDC 75017 produced high levels of GABA and exhibited anti-inflammatory, cholesterol-lowering and antioxidant properties (23–25). In the present study, the potential protective effect of *L. plantarum* NDC 75017 was investigated through the establishment of a D-gal-induced aging model in rats. The behavioral changes were examined and the ATP levels, mitochondrial function and mitochondrial ultrastructural changes in the cerebral cortical neurons of the rats were examined to further investigate the potential mechanism underlying the neuroprotective effect of *L. plantarum* NDC 75017.

## Materials and methods

### Materials

D-gal, rhodamine 123 (Rh123), rotenone and 4-(2-hydroxyethyl)-1-piperazineethanesulfonic acid (HEPES) were purchased from Sigma-Aldrich (St. Louis, MO, USA). Bicinchoninic acid protein and ATP assay kits were purchased from Wuhan Boster Bioengineering Co. Ltd. (Wuhan, China) and Beyotime Institute of Biotechnology (Beijing, China), respectively. All other chemicals used were of the highest quality that is commercially available. The JSM25610LV transmission electron microscope used in the study was produced by Japan Electron Optics Laboratory Co., Ltd. (Tokyo, Japan).

### Lactobacillus strain and growth conditions

The *L. plantarum* NDC 75017 were isolated from a traditional Chinese fermented yogurt (from the Tongliao range of Inner Mongolia, China). The bacteria were anaerobically grown at 30°C overnight in de Man-Rogosa-Sharpe broth (Difco^™^, Beckman-Coulter, Miami, FL, USA). The bacterial cells were collected by centrifugation at 8,000 × g for 5 min, washed three times with phosphate-buffered saline and adjusted to 1×10^8^, 1×10^9^ and 1×10^10^ CFU/ml for oral administration to the rats.

### Animals and experimental design

A total of 50 male Wistar rats (weighing 180–200 g) were obtained from the Vital River Laboratory Animal Technology Co., Ltd. (Beijing, China). The rats were housed in separate cages and had free access to food and water for ≥1 week to acclimate prior to the initiation of treatment. The animals were housed in a limited-access animal facility where the room temperature and relative humidity were set to 22±2°C and 55±10%, respectively. Artificial lighting provided a 24-h cycle of 12-h light/12-h dark (light from 7:00 a.m. to 7:00 p.m.). All of the animal experiments were approved by the Animal Care and Use Committee of Heilongjiang Province, China. After the one-week acclimation period, the rats were randomly divided into five groups, with 10 rats in each group. The rats were orally administered 1 ml/100 g body weight of different concentrations (CFU/ml) of *L. plantarum* once per day for 49 days (seven weeks). The rats in the control and aging model groups were only administered a vehicle (0.9% saline) or D-gal, respectively. The treatments were as follows: Group I, 0.9% normal saline (control group); Group II, D-gal (100 mg/kg) subcutaneously (D-gal group); Group III, low-dose *L. plantarum* [1×10^8^ CFU/100 mg, per oral (p.o.)] plus D-gal (100 mg/kg) (L + D-gal group); Group IV, medium-dose *L. plantarum* (1×10^9^ CFU/100 mg, p.o.) plus D-gal (100 mg/kg) (M + D-gal group); Group V, high-dose *L. plantarum* (1×10^10^ CFU/100 mg, p.o.) plus D-gal (100 mg/kg) (H + D-gal group).

### Water maze test

Spatial learning was investigated after the seven weeks of D-gal injection using the Morris water escape task according to a previous study (26). From the 44th day, the rats were trained for four days until the 49th day, when the time taken to climb onto the platform (escape latency) was recorded for each rat.

### Observation of mitochondrial ultrastructure

After seven weeks of treatment, the mitochondrial ultrastructure of the rat cerebral cortices was observed using a transmission electron microscope as previously described (27). A total of 15 rats (n=3 from each group) were sacrificed through an intraperitoneal injection of an overdose of sodium pentobarbital (80 mg/kg). The cerebral cortices were isolated, fixed and perfused with 2.5% glutaraldehyde. The cortices were subsequently stored overnight at 4°C. Following post-fixation in 2% osmium tetroxide for 2 h at 4°C, the tissues were dehydrated in an ascending graded ethanol and acetone series and immersed in an acetone/Epon 812 mixture at ratios of 1:1, 1:2 and 1:3 for 0.5, 2 and 10 h, respectively. Ultra thin sections (70 nm) were prepared, counterstained with uranyl acetate and lead citrate and examined using the JSM25610LV transmission electron microscope.

### Isolation and purification of mitochondria

Mitochondria were isolated from the cerebral cortices of the rats through homogenization and differential centrifugation according to the methods performed in a previous study (28). The protein content of the isolated mitochondria samples was determined using the Bradford protein assay. Bovine serum albumin was used to construct a standard curve.

### Determination of ATP content

The levels of ATP in the mitochondria were measured using the ATP Bioluminescence Assay kit (Beyotime Institute of Biotechnology) according to the manufacturers’ instructions. Briefly, the levels of ATP were determined by mixing 50 μl mitochondrial solution with 50 μl luciferase solution, which catalyzes ATP-mediated light production from luciferin. The amount of emitted light, measured using a microplate luminometer (Promega, Madison, WI, USA), was linearly associated with the ATP concentration.

### Measurement of the mitochondrial permeability transition (MPT)

The MPT value was determined using an ultraviolet spectrophotometer to measure the absorbance at 540 nm (A_540 nm_), as previously described (29). Briefly, the isolated mitochondria were diluted to 0.5 mg/ml and incubated in the assay buffer (125 mm sucrose, 65 mm KCl, 5 mm succinate, 5 mm rotenone and 10 mm Tris-HCl; pH 7.4). MPT was initiated and monitored prior to and following the addition of 50 μM calcium chloride for 5 min. The results were expressed as the decrease in the absorbance at 540 nm.

### Detection of mitochondrial membrane potential (Δψ_m_)

Δψ_m_ was detected according to the methods of a previous study (29), with modifications. Briefly, fluorescence (excitation at 503 nm and emission at 527 nm) occurring in the reaction buffer (250 mm sucrose, 2 mm HEPES, 0.5 mm KH_2_PO_4_, 4.2 mm sodium succinate at pH 7.4 and 0.3 mm Rh123) was measured using an F-4500FL spectrophotometer (Hitachi High-Technologies Co., Tokyo, Japan). The diluted mitochondria (0.5 mg/ml) were added to the buffer and incubated for 3 min. The fluorescence was measured again using the F-4500FL spectrophotometer. Finally, the change in Δψ_m_ was expressed by the decrease in fluorescence.

### Activities of the mitochondrial respiratory chain

MTT reduction was used to assess the activities of the mitochondrial respiratory chain. The methods and procedures utilized in the present study were identical to those of a previous study (28). Briefly, 0.02 ml MTT (0.1 mg/ml) was added to the mitochondrial solution containing 60 μg protein. The reaction mixture was co-incubated at 37°C for 30 min and centrifuged at 1,000 g for 5 min at room temperature. The obtained pellet was dissolved in 1 ml of acidic isopropanol and re-centrifuged at 1,000 g for 5 min at room temperature to obtain the supernatant. The absorbance was measured at 595 nm and the results were presented as A595 nm/mg protein.

### Statistical analysis

Data are presented as the mean ± standard deviation. The data were analyzed using one-way analysis of variance followed by least significant difference post hoc tests to compare the different treatment groups. P<0.05 was considered to indicate a statistically significant difference.

## Results

### Effect of L. plantarum NDC 75017 on the spatial learning of aging rats induced by D-gal

**Figure 1:**
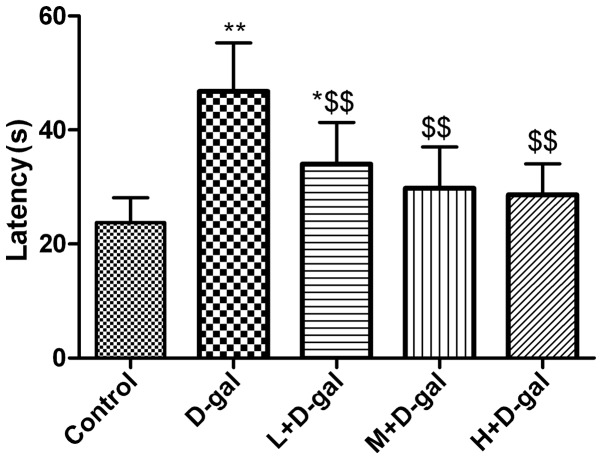
Effect of *Lactobacillus plantarum* NDC 75017 on the spatial learning of D-gal-induced aging rats. Data are presented as the mean ± standard deviation (n=10). ^*^P<0.01 and ^**^P<0.01 vs. the control group; ^$$^P<0.01 vs. the D-gal group. L, low-dose *L. plantarum* NDC 75017; M, medium-dose *L. plantarum* NDC 75017; H, high-dose *L. plantarum* NDC 75017; D-gal, D-galactose.

From the 44th day, the rats were trained for four days, following which the water maze test was carried out. The escape times of the rats in D-gal group were significantly higher (P<0.01) compared with those of the control group rats. However, the escapes times of rats in the L + D-gal, M + D-gal and H + D-gal groups were significantly lower (P<0.01) compared with those of rats in the D-gal group (Fig. 1).

### D-gal-induced ultrastructural changes in neuronal mitochondria and the effect of L. plantarum NDC 75017

**Figure 2:**
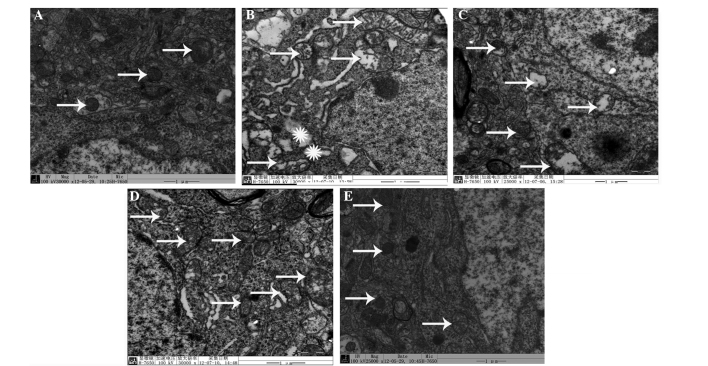
Representative changes in the mitochondrial ultrastructure in the rat cerebral cortex (n=3). (A) Control; (B) aging model (D-gal); and (C–E) low-, medium- and high-dose *Lactobacillus plantarum* + D-gal groups, respectively. The black arrows and stars represent the mitochondria and endoplasmic reticulum, respectively. D-gal, D-galactose.

Ultrastructural changes in the mitochondria of the cerebral cortical neurons were observed and the results are shown in Fig. 2. After seven weeks of D-gal treatment, marked pathological changes were observed in the mitochondria of the cerebral cortical neurons of the D-gal-induced aging group compared with the control group, including rupture and scarcity of the cristae and vacuolization (Fig. 2B). Coadministration of *L. plantarum* NDC 75017 and D-gal decreased the neuronal mitochondria injury in rats with dose-dependent effects (Fig. 2C–E).

### Effect of L. plantarum NDC 75017 on the D-gal-induced changes in ATP content

**Figure 3:**
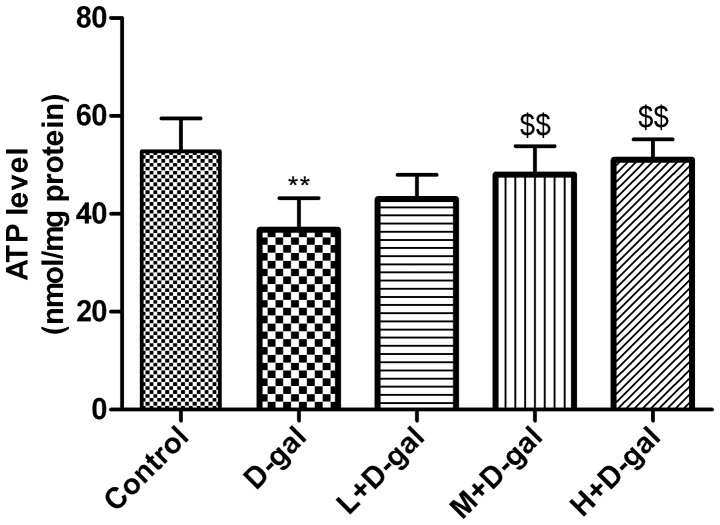
Effect of *Lactobacillus plantarum* NDC 75017 on the D-gal-induced the changes in ATP content. Data are presented as the mean ± standard deviation (n=10). ^**^P<0.01 vs. the control group; ^$$^P<0.01 vs. the D-gal group. L, low-dose *L. plantarum* NDC 75017; M, medium-dose *L. plantarum* NDC 75017; H, high-dose *L. plantarum* NDC 75017; D-gal, D-galactose; ATP, adenosine triphosphate.

The levels of ATP in the mitochondria in the cerebral cortical neurons of the rats were determined. The results are shown in Fig. 3. Compared with the control group, the levels of ATP in the D-gal group were significantly lower (P<0.01). However, significant increases in the levels of ATP were observed in the M and H + D-gal groups (P<0.01) compared with the D-gal group.

### Effect of L. plantarum NDC 75017 on Ca^2+^-induced changes in MPT

**Figure 4:**
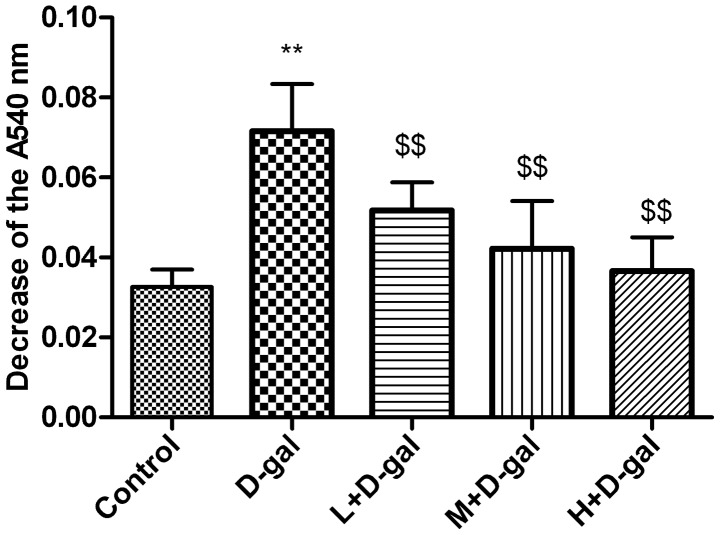
Effect of *Lactobacillus plantarum* NDC 75017 on Ca^2+^-induced changes in mitochondrial permeability transition in a D-gal-induced model of aging. Data are presented as the mean ± standard deviation (n=10). ^**^P<0.01 vs. the control group; ^$$^P<0.01 vs. the D-gal group. L, low-dose *L. plantarum* NDC 75017; M, medium-dose *L. plantarum* NDC 75017; H, high-dose *L. plantarum* NDC 75017; D-gal, D-galactose.

Ca^2+^-induced changes in MPT were assessed and the results are shown in Fig. 4. The MPT in the mitochondria of rat cerebral cortical neurons was significantly higher (P<0.01) in the D-gal group compared with that in the control group. However, the MPT in the L, M and H + D-Gal groups was significantly lower than that in the D-gal group, with decreases of 27.7, 41.1, and 48.9%, respectively, relative to the D-gal model group (all P<0.01).

### Effect of L. plantarum NDC 75017 on D-gal-induced changes in Δψ_m_

**Figure 5:**
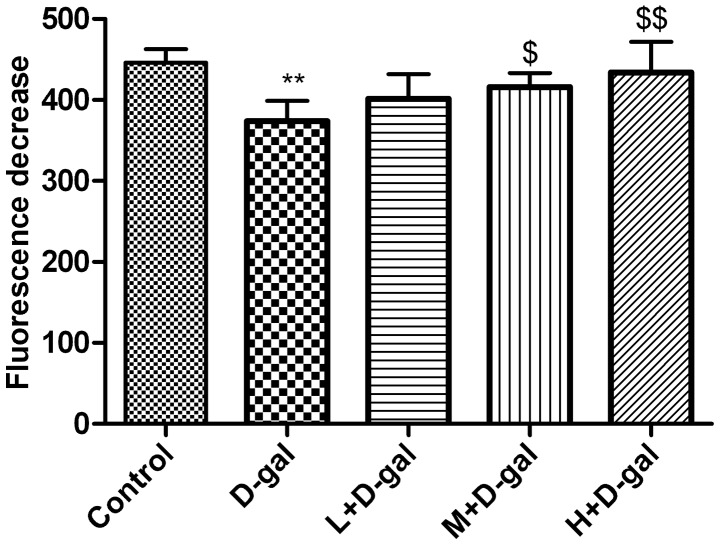
Effect of *Lactobacillus plantarum* NDC 75017 on the D-gal-induced changes in mitochondrial membrane potential. Data are presented as the mean ± standard deviation (n=10). Statistical significance was determined using one-way analysis of variance. ^**^P<0.01 vs. the control group; ^$^P<0.05 and ^$$^P<0.01 vs. the D-gal group. L, low-dose *L. plantarum* NDC 75017; M, medium-dose *L. plantarum* NDC 75017; H, high-dose *L. plantarum* NDC 75017; D-gal, D-galactose.

Δψ_m_ was determined by monitoring the dynamic fluorescence quenching of Rh123. The initial fluorescence (714±36) was markedly reduced following the addition of mitochondria. As shown in Fig. 5, mitochondria of the rats in the control group quenched the fluorescence to 446±17, and the extent of quenching exhibited by mitochondria in the D-gal group was significantly lower (374±25) (P<0.01). In the M and H + D-gal groups, fluorescence quenching was significantly increased to 416±17 and 434±38, respectively (P<0.05 and P<0.01, respectively) compared with the D-gal group (Fig. 5).

### Effect of L. plantarum NDC 75017 on the D-gal-induced changes in the activity of the mitochondrial respiratory chain

**Figure 6:**
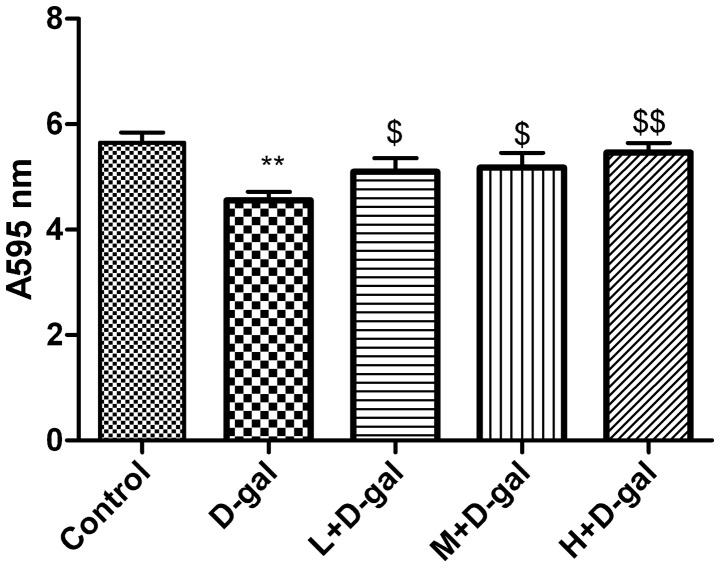
Effect of *Lactobacillus plantarum* NDC 75017 on the activities of the mitochondrial respiratory chain. The activity of mitochondrial succinate dehydrogenase is expressed as A_595 nm_/(mg protein). Data are presented as the mean ± standard deviation (n=10). ^**^P<0.01 vs. the control group; ^$^P<0.05 and ^$$^P<0.01 vs. the D-gal group. L, low-dose *L. plantarum* NDC 75017; M, medium-dose *L. plantarum* NDC 75017; H, high-dose *L. plantarum* NDC 75017; D-gal, D-galactose.

The reduction of the water-soluble tetrazolium salt MTT to formazan is regarded as an indicator of mitochondrial respiration, particularly the activity of mitochondrial succinate dehydrogenase. Compared with the control group, mitochondrial enzymatic activity was significantly lower (P<0.01) in the D-gal group, and progressive improvements were observed in the L, M and H + D-gal groups (Fig. 6).

## Discussion

Several theories have been proposed to explain age-related mitochondrial dysfunction. The oxidative stress theory is the most important theory of aging proposed in past few decades (30). The targeted accumulation of ROS damages the mitochondria, which are more sensitive and vulnerable to oxidative stress than other organelles in cells due to their structural and functional characteristics (6). The decrease in mitochondrial function and the increase in mitochondrial DNA damage suggests that the progressive accumulation of oxidative DNA damage is a contributing factor to cell apoptosis or necrosis, with the generation of more ROS during aging (10,20,28,29). Chronic administration of D-gal has been widely used to mimic the process of brain aging. The D-gal-induced model of brain aging is important in the development of suitable anti-aging drug strategies (31,32). A number of studies have revealed that low doses of D-gal (such as 50 or 100 mg/kg) decrease the learning and memory abilities of mice and rats, as demonstrated through the T-maze, Y-maze and Morris water maze tests (26,33). In the present study, rats administered 100 mg/kg D-gal for seven weeks exhibited a significant decrease in learning and memory ability, as confirmed through their performance in the Morris water maze test.

At present, mitochondrial Ca^2+^ homeostasis is the center of widespread interest in scientific studies due to its modulatory role in numerous physiological processes and its involvement in cell death (6,28,29). Ca^2+^ uptake and release from the mitochondrial membrane via a variety of mechanisms control the local regulation of intracellular Ca^2+^ concentration. The mitochondrial Ca^2+^ dysfunction can cause MPT pore opening, leading to a change in the Δψ_m_ and resulting in mitochondrial swelling and dysfunction, and even cell death (28). It has been demonstrated that injecting rodents with D-gal for 6–10 weeks induces aging, affecting mitochondrial bioenergetics. This leads to the activity of the electron transport chain complex becoming compromised and a decrease in the rate of ATP synthesis (6,28). The results of the present study indicated that chronic administration of D-gal impaired the activity of the mitochondrial enzyme complex, Δψ_m_, mitochondrial membrane permeability and ATP production ability. These changes were alleviated with the administration of *L. plantarum* NDC 75017, which may be associated with its antioxidative properties.

GABA supplementation may activate the upstream signal survival pathways that regulate mitochondrial function, such as the phosphoinositide 3-kinase-Akt signal survival pathway (34). GABA also plays an important role in the aging process and diseases including PD and HD (22,34,35). GABA increases the circulation of serum lipids and decreases mitochondrial injury mediated by oxidative stress (21,36). Certain LABs have been demonstrated to directly regulate GABA in the microbiome-gut-brain axis in mice (22). *L. plantarum* NDC 75017 has the ability to produce high levels of GABA *in vitro*, which may contribute to its anti-aging and D-gal-induced mitochondrial dysfunction-alleviating abilities. The precise mechanisms for this should be investigated in further studies.

In conclusion, the results of the present study revealed that *L. plantarum* NDC 75017 was able to alleviate learning and memory-associated injuries in aging rats by reducing mitochondrial dysfunction induced by D-gal. This may be associated with its antioxidant and GABA-producing activities.
